# The Effect of Ondansetron and Dexamethasone on Nausea and Vomiting under Spinal Anesthesia

**Published:** 2017-01

**Authors:** Navid Kalani, Hasan Zabetian, Mohammad Sadegh Sanie, Mansour Deylami, Mohammad Radmehr, Reza Sahraei, Hossein Kargar Jahromi, Wesam Kooti

**Affiliations:** 1 Medical Ethic Research Center, Jahrom University of Medical Sciences, Jahrom, Iran;; 2 Research Center For Non-Communicable Diseases, Jahrom University of Medical Sciences, Jahrom, Iran;; 3 Department of Anesthesiology, Jahrom University of Medical Sciences, Jahrom, Iran;; 4 Department of Anesthesiology, Golestan University of Medical Sciences, Golestan, Iran;; 5 Student Research Committee, Kurdistan University of Medical Sciences, Sanandaj, Iran

**Keywords:** Ondansetron, Dexamethasone, Nausea, Vomiting; Surgery, Spinal anesthesia

## Abstract

**BACKGROUND:**

During abdominal surgery under regional anesthesia, nausea may happen due to several contributing factors. This study compared the effects of ondansetron and dexamethasone on nausea and vomiting under spinal anesthesia.

**METHODS:**

One hundred and twenty patients of 15 to 35 years old with ASA class I and II were enrolled. Before administering either ondansetron or dexamethasone, blood pressure and heart rate of the patients were recorded. The patients received 70 mg of 5% lidocaine for spinal anesthesia. Patients who received 6 mg of ondansetron were considered as group A, while group B received 8 mg of dexamethasone. The level of nausea and vomiting, blood pressure, heart rate and respiratory rate of each patient was measured at 1, 5, 10, 15 and 30 minutes after spinal anesthesia and during recovery (every 5 minutes).

**RESULTS:**

There was a significant difference between nausea and vomiting between the two groups after spinal anesthesia within the first and fifth minutes. There was no significant difference between nausea and vomiting between the two groups within 10, 15 and 30 minutes and during recovery at 5, 10, 15 and 30 minutes.

**CONCLUSION:**

Dexamethasone and ondansetron were shown to equally reduce the incidence of nausea and vomiting under spinal anesthesia and can be recommended as a good choice for prevention of nausea and vomiting during surgeries.

## INTRODUCTION

Worldwide, one of the most common obstetrics surgeries is caesarean section,^1^ while spinal anesthesia is used for caesarean sections as a safe, easy and quick technique.^2^ Nausea and vomiting after caesarean sections have been reported in more than 66% of cases,^3^^,^^4^ due to sudden contractions in diaphragm and manipulation and stretching of the abdominal viscera.^5^ Spinal anesthesia in a cesarean delivery can prevent the risk of pulmonary aspiration that may occur under general anesthesia.^5^

Stimulation of pharyngeal reflex can be noticed in abdominal surgeries, physical rupture and manipulation of abdominal viscera due to the release of humoral HT-5 substances, which trigger the HT3-5 receptors on vagal afferent neurons.^3^ During abdominal surgery under regional anesthesia, nausea may happen due to several contributing factors such as sympathetic blocks followed by parasympathetic dominance which is the most important cause of nausea after spinal anesthesia, hypotension, decreased perfusion of central nervous system, psychological changes (anxiety), and sudden abdominal movements during surgery and prescription of drugs.^6^

The risk of nausea and vomiting in final stages of pregnancy is higher due to hormonal changes and increased intra-abdominal pressure.^6^ The major complications of spinal anesthesia were shown to be nausea and vomiting, leading to an unpleasant experience in these patients.^7^ In recent decade, spinal anesthesia has been applied as a safe and fast method for cesarean delivery.^2^^,^^7^ In many cases in Iran, caesarean section is carried out as emergency without prior preparation, where the patients are usually not fasting during surgery. Hence, spinal anesthesia in such cases is regarded as a standard method to reduce the risk of vomiting and aspiration.^3^^,^^4^^,^^8^

One of the most common problems with spinal anesthesia is the incidence of vomiting as the uterus is pushed back into the abdominal area, accounting for 66% of cases^3^^,^^4^^,^^8^ which is is dealt by a variety of medications, among which metoclopramide is the most common.^9^^-^^11^ However, there are extrapyramidal complications to be associated with the drug, leading to much concern and caution when prescribing metoclopramide.^9^^-^^11^

Dexamethasone has been introduced an an inexpensive and widely available drug to control nausea and vomiting.^12^^,^^13^ Similarly, ondansetron is considered as an effective drug for prevention and treatment of nausea and vomiting that is well tolerated by the patients.^6^ This drug is applied in surgeries which may be accompanied by nausea and vomiting. The serotonin receptor antagonists (HT3-5) and dexamethasone were reported as ideal medications for controlling nausea and vomiting, without any adverse side effects.^14^^-^^17^

The exact mechanism of dexamethasone in preventing nausea and vomiting is still unknown, but it may be due to inhibition of prostaglandin synthesis.^14^^,^^15^ Moreover, ondansetron can inhibit serotonin receptor leading to the prevention of nausea and vomiting.^15^^-^^17^ Given the high prevalence of cesarean section in Iran, the harmful effects of nausea and vomiting during and after operation and insufficient studies on the effect of dexamethasone and ondansetron for prevention of nausea and vomiting during spinal anesthesia, this study intended to compare the effect of dexamethasone and ondansetron in controlling of nausea and vomiting during spinal anesthesia.

## MATERIALS AND METHODS

In a double-blind, randomized, controlled trial study, 120 patients of 15 to 35 years old with ASA class I and II (American Society of Anesthesia Score) were enrolled. The samples were selected through a simple random sampling method. The study population compromised patients undergoing surgery through spinal anesthesia and were admitted in Motahari Hospital in Jahrom, Iran. 

The patients were randomly divided into two equal groups of 60 subjects. Before entering the operation room, the patients did not receive any premedication drugs. Before inclusion in the study, the patients were informed about the purpose of the study and potential complications and an informed written consent was provided from each participant. The study was approved in institution ethics committee.

Inclusion criteria were being pregnant and age of 15 to 35 years old, diagnosis with ASA class I and II, to be candidate to undergo caesarean section, having no history of known physical and mental illnesses, and any history of taking pain killers and anti-depressants, sleeping pills and psychotropics. The exclusion criteria were weighing more than 100 kg, age over 35 years or less than 15 years, history of drug or alcohol dependence, treatment with antidepressants, sleeping pills and psychotropic, lack of appropriate communication with patients for evaluating postoperative nausea and vomiting, need for hospitalization in the ICU after surgery, any previous history of allergy to ondansetron or dexamethasone, need for additional treatment and sickness during surgery, rising level of anesthesia and reduction or loss of respiration, hemodynamic disorder and patient dissatisfaction. 

Both groups of patients were hydrated with 7 ml/kg of Ringer’s solution. Before administering either substances (ondansetron or dexamethasone), blood pressure and heart rate of the patients were recorded. The patients received 70 mg of 5% lidocaine for spinal anesthesia. Group A received 6 mg of ondansetron, while group B received 8 mg of dexamethasone (prior to clamping the cord) and in group B, 8 mg dexamethasone was administered at the same time (before clamping the umbilical cord). 

The levels of nausea and vomiting, blood pressure, heart rate and respiratory rate of each patient were measured at 1, 5, 10, 15 and 30 minutes after spinal anesthesia and during recovery (every 5 minutes based on the number of vomiting, retching or nausea told by the patient). Ephedrine was applied if hypotension occurred during the operation. At the end of surgery, the patient was transferred to the recovery room. The minimum recovery time was one hour. Neither the patient nor the nurse responsible for postoperative follow-up were aware of the primary drugs prescribed to patients. Data were analyzed using SPSS software (version 11, Chicago, IL, USA). Fisher and Chi-square tests were performed for comparison of groups. A p value less than 0.05 was considred statistically significant.

## RESULTS

There was a significant difference between nausea and vomiting between the two groups of ondansetron and dexamethasone after spinal anesthesia within the first and fifth minutes (*p*<0.05, Table 1). There was no statistically significant difference between nausea and vomiting between the two groups within 10, 15 and 30 minutes (*p*>0.05, Table 1).

**Table 1:** The frequency of nausea and vomiting in two ondansetron and dexamethasone groups at 1, 5, 10, 15 and 30 minutes after spinal anesthesia

**Minute**	**Group**	**Nausea and vomiting**		***p*** ** value**
		**Yes**	**No**	
First	Ondansetron	0	60(100%)	0.027*
	Dexamethasone	6 (10.2%)	54 (89.8%)	
5	Ondansetron	0	60 (100%)	0.006*
	Dexamethasone	8 (13.6%)	52 (86.4%)	
10	Ondansetron	6 (10.2%)	54 (89.8%)	0.272*
	Dexamethasone	2 (3.4%)	58 (96.6%)	
15	Ondansetron	4 (6.7%)	56 (93.3%)	0.142**
	Dexamethasone	9 (15.3%)	51 (84.7%)	
30	Ondansetron	10 (16.7%)	50 (83.3%)	0.040**
	Dexamethasone	3 (5.1%)	57 (94.9%)	

* Fisher’s Exact Test,

** Chi-Square Test

During 1, 5, 10, 15 and 30 after spinal anesthesia, 6 (10.2%), 8 (13.6%), 2 (3.4%), 9 (15.3%) and 3 patients (5.1%) in the dexamethasone group experienced nausea and vomiting, respectively, while these figures ondansetron group experiencing nausea and vomiting were 6 (10%), 4 (6.7%) and 10 patients (16.7%), repectively. There was no significant difference between nausea and vomiting between the two groups of ondansetron and dexamethasone during recovery at 5, 10, 15 and 30 minutes (p value> 0.05, Table 2).

**Table 2:** The frequency of nausea and vomiting in two ondansetron and dexamethasone groups at 5, 10, 15 and 30 minutes on recovery

**Minute**	**Group**	**Nausea and vomiting**		***p*** ** value**
		**Yes**	**No**	
5	Ondansetron	4 (6.7)	56 (93.3%)	0.658**
	Dexamethasone	5 (13.6%)	95 (86.4%)	
10	Ondansetron	4 (10.2%)	56 (89.8%)	0.119*
	Dexamethasone	0	60 (100%)	
15	Ondansetron	3 (5.1%)	57 (94.9%)	0.619*
	Dexamethasone	1 (1.7%)	59 (98.3%)	
30	Ondansetron	4 (10.2%)	56 (89.8%)	0.119*
	Dexamethasone	0	60 (100%)	

* Fisher’s Exact Test,

** Chi-Square Test

In the recovery room at 5, 10, 15 and 30 minutes, 3 (5%), 2 (3.4%), and 1 patient (1.7%) in the dexamethasone group experienced nausea and vomiting, respectively , while the figures for ondansetron group experiencing nausea and vomiting in the recovery room were 4 (6.7%), 4 (6.7%) and 3 patients (6.7%), respectively. From 30 minutes onwards in the recovery, i.e. at 35, 40, 45, 50, 55 and 60 minutes, none of the patients in ondansetron and dexamethasone groups experienced nausea and vomiting.

There was no statistically significant difference between the mean levels of heart rate, systolic and diastolic blood pressures, respiration and oxygen saturation among both groups of ondansetron and dexamethasone during the recovery and after spinal anesthesia (*p*>0.05, Table 3). None of the patients in both groups had dysrhythmia or bradycardia. A total of 11 (9.1%) patients in both groups had systolic hypotension. Eight (6.6%) patients in the two groups experienced diastolic hypotension. None of the patients in the two groups experienced increase in respiratory rate, oxygen saturation drop and oxygen desaturation drop after spinal anesthesia.

**Table 3:** Ondansetron and dexamethasone vital signs in both groups during recovery and after spinal anesthesia

**Group**	**Group**		***p*** ** value**
	**Ondansetron**	**Dexamethasone**	
Heart rate	71.06±4.91	71.13±4.86	0.062
Systolic blood pressure	125.86±4.98	125.60±5.07	0.064
Diastolic blood pressure	79.28±3.26	78.93±3.54	0.06
Respiration	12±2.7	12±2.2	0.19
Oxygen saturation	94%±2	96%±2.9	0.28

## DISCUSSION

Our findings demonstrated that 6 mg of ondansetron and 8 mg of dexamethasone could equally curtail the incidence of nausea and vomiting in patients undergoing surgery through spinal anesthesia. In some countries including the United States, spinal anesthesia is the method of choice for elective emergency caesarean surgeries, whereas certain hospitals adopt this method in 41% of patients.^18^

The effects of spinal anesthesia in pregnant and non-pregnant women vary. Distribution of anesthetic drug into the cerebrospinal fluid in pregnant women is less predictable, which is associated not only to increased pressure on the spinal canal,^19^ but also to a series of successive changes in the balance of acids and bases^20^ and cerebrospinal fluid protein contents^21^ due to physiological changes during pregnancy. In addition, the side effects of spinal anesthesia, such as hypotension, nausea and vomiting, hypersensitivity to intrathecal opioids is more common in pregnant women compared to non-pregnant women.^22^

In this study, a dose of 6 mg of ondansetron was chosen because is as effective in the prevention and treatment of nausea and vomiting after surgery similar to the higher dose. Moreover, there will not be any side effects at this dosage.^23^ Pearman *et al.* suggested that the effect of 6 mg ondansetron might be more effective than 4 mg ondansetron in pregnant women who are more prone to nausea and vomiting.^24^ Borgeat *et al.* found out the direct therapeutic and anti-nausea effect of sub-hypnotic doses of propofol in gynecological, gastroenterological and orthopedic surgeries. Nevertheless, subsequent studies revealed that propofol can prevent nausea and vomiting in elective cesarean section surgery under spinal anesthesia.^25^

In a study by Szarvas *et al.*, the incidence of nausea and vomiting in the first 24 hours after injection of intrathecal morphine was 70, 73 and 72 percent, respectively.^26^ Pirat *et al. *showed that 8 mg of oral ondansetron and 4 mg of intravenous ondansetron did not prevent nausea and vomiting caused by intrathecal meperidine during surgery.^27^ In another study (2009), the HT3-5 receptor antagonists were effective in prevention of nausea and vomiting caused by intrathecal morphine in women undergoing cesarean section.^28^ In a study by Nortcliffe *et al.*, dexamethasone was not effective in preventing nausea and vomiting caused by intrathecal morphine.^29^

In addition, the study by Wu *et al.* indicated that dexamethasone was not effective in the prevention of nausea and vomiting caused by intrathecal morphine.^30^ The study by Tzeng *et al.* suggested that dexamethasone could curtail nausea and vomiting caused by opidural morphine in cesarean section.^31^ Movafegh *et al.* showed that 8 mg of dexamethasone could effectively reduce nausea and vomiting caused by intrathecal meperidine.^32^ The reason for such inconsistency of results in the prevention of nausea and vomiting can be hormonal changes, gender, age, weight, pain, type of surgery, duration of surgery, history of nausea and vomiting after surgery, intrathecal drug dose and type and dose of HT3-5 antagonists and dexamethasone.

This study demonstrated that 8 mg of dexamethasone and 6 mg of ondansetron were equally able to prevent intrathecal nausea and vomiting after surgery. Considering our findings, dexamethasone can be regarded as a safe alternative to ondansetron for prevention of nausea and vomiting after surgery.
